# Chitosan Modification of Adenovirus to Modify Transfection Efficiency in Bovine Corneal Epithelial Cells

**DOI:** 10.1371/journal.pone.0012085

**Published:** 2010-08-10

**Authors:** I-Jong Wang, Min-Chao Jhuang, Yu-Hsin Chen, Lung-Kun Yeh, Chia-Yang Liu, Tai-Horng Young

**Affiliations:** 1 Department of Ophthalmology, National Taiwan University Hospital, College of Medicine, National Taiwan University, Taipei, Taiwan; 2 Institute of Polymer Science and Engineering, National Taiwan University, Taipei, Taiwan; 3 Department of Ophthalmology, Chang-Gung Memorial Hospital, Chang-Gung University College of Medicine, Linko, Taiwan; 4 Department of Ophthalmology and Cell Biology, Neuroscience and Anatomy, University of Cincinnati College of Medicine, Cincinnati, Ohio, United States of America; 5 Institute of Biomedical Engineering, College of Medicine and College of Engineering, National Taiwan University, Taipei, Taiwan; Johns Hopkins School of Medicine, United States of America

## Abstract

**Background:**

The purpose of this study is to modulate the transfection efficiency of adenovirus (Ad) on the cornea by the covalent attachment of chitosan on adenoviral capsids via a thioether linkage between chitosan modified with 2-iminothiolane and Ad cross-linked with N-[γ-maleimidobutyryloxy]succinimide ester (GMBS).

**Methodology/Principal Findings:**

Modified Ad was obtained by reaction with the heterobifunctional crosslinking reagent, GMBS, producing maleimide-modified Ad (Ad-GMBS). Then, the chitosan-SH was conjugated to Ad-GMBS via a thioether bond at different ratios of Ad to GMBS to chitosan-SH. The sizes and zeta potentials of unmodified Ad and chitosan-modified Ads were measured, and the morphologies of the virus particles were observed under transmission electron microscope. Primary cultures of bovine corneal epithelial cells were transfected with Ads and chitosan-modified Ads in the absence or presence of anti-adenovirus antibodies. Chitosan modification did not significantly change the particle size of Ad, but the surface charge of Ad increased significantly from −24.3 mV to nearly neutral. Furthermore, primary cultures of bovine corneal epithelial cells were transfected with Ad or chitosan-modified Ad in the absence or presence of anti-Ad antibodies. The transfection efficiency was attenuated gradually with increasing amounts of GMBS. However, incorporation of chitosan partly restored transfection activity and rendered the modified antibody resistant to antibody neutralization.

**Conclusions/Significance:**

Chitosan can provide a platform for chemical modification of Ad, which offers potential for further *in vivo* applications.

## Introduction

Gene delivery to the three major cell types of the cornea using viral and non-viral methods has been demonstrated *in vivo* and *in vitro* [1]. Viral and non-viral vectors have their own advantages and limitations. For example, recombinant adenovirus (Ad) has been extensively evaluated in gene delivery to the cornea in many studies [2]–[8]. This method can achieve a high level of transgene expression, but immune responses may be raised against Ad proteins, thereby limiting further administration of the virus. In contrast, non-viral cationic polymer vectors can bind to the negatively charged cell surface, but their application in corneal gene delivery may be limited by cytotoxicity and low infection efficiency.

Various approaches have been developed to modify Ad tropism. New generations of Ad vectors have been designed to lower their immunogenicity. In one approach, the capsid proteins of Ad are chemically modified with polymers. Modification of Ad vectors with polyethylene glycol (PEG) prolongs extended circulation kinetics in murine models and circumvents neutralization of the Ad vectors by antibodies. The activated PEG reacts preferentially with the ε-amino termini of lysine residues on the capsid, specifically the hexon, fiber, and penton base proteins [9]–[14]. Furthermore, PEGylated Ad vectors attenuate the ability of the vector to be taken up by antigen-presenting cells, thereby reducing inflammatory responses. Animals administered PEGylated Ad vectors exhibit reduced levels of both cell-mediated and humoral immune responses, resulting in significant transgene expression on re-administration of unmodified Ad vectors in the lung [15], [16]. However, PEGylation of Ad vectors leads to reduced infectivity due to steric hindrance from flexible PEG chains [9]–[11], [14]–[18]. Ad vectors coated with polymers other than PEG have also been developed. Seymour *et al.* used a multivalent hydrophilic polymer based on poly[*N*-(2-hydroxypropyl)methacrylamide] (pHPMA) to modify Ad vectors [19]–[22]. These modified vectors also show extended plasma circulation time, reduced toxicity, and evasion of neutralizing antibodies.

Chitosan has been proposed for parenteral drug delivery and mucosal drug delivery due to its unique biological properties, such as the ability to adhere to mucus, to permeate mucosal barriers, and to be biodegraded in the rich lysozyme-containing mucus [23], [24]. These features are important for the application of chitosan to the treatment of ocular surface diseases. Some studies examining gene delivery to the cornea and ocular surface have demonstrated enhancement of adenoviral infectivity by using a noncovalent complex consisting of chitosan [25], [26]; however, chitosan has not yet been used for the chemical modification of Ad.

The purpose of this study was to covalently modify a recombinant Ad encoding a transgene with chitosan, to shield the vector from recognition by anti-Ad antibodies, and to ablate the pathways that Ad normally activates. Chitosan, a cationic natural polymer, was chosen to modify adenoviral capsids because it would change the Ad surface charge from negative to neutral or positive, facilitating attachment of the modified Ad to the negatively charged cell membrane.

## Materials and Methods

### Biological and chemical reagents

Most culture reagents, including Dulbecco's Modified Eagle Medium (DMEM)/F-12 (Ham) 1×, L-glutamine, HEPES, DMEM, D-glucose, sodium pyruvate, TrypLETM Express, phenol red, fetal bovine serum, *Escherichia coli* LPS, penicillin-streptomycin (10,000 U/mL), fungizone antimycotic, amphotericin B (250 µg/mL), insulin-transferrin-selenium supplement, and gentamicin solution, were purchased from Invitrogen (Carlsbad, CA). Chemical reagents used in this study included Dispase II (Roche, Mannheim, Germany), rabbit polyclonal antibody to Ad type 5 (Abcam, Cambridge, UK), chitosan oligosaccharide lactate, (M_n_<5,000, Sigma-Aldrich, St. Louis, MO), 2-iminothiolane hydrochloride (Sigma-Aldrich), N-[γ-maleimidobutyryloxy]succinimide ester (GMBS, Pierce, Rockford, IL), phosphate-buffered saline (PBS), DL-dithiothreitol (DTT, Sigma-Aldrich), and 5,5′-dithiobis-(2-nitrobenzoic acid) (DTNB, MP Biomedicals, Solon, OH).

### Primary culture of bovine corneal epithelial cells

The methods used for primary culture of bovine corneal epithelial cells were described previously [27], [28]. Briefly, eyes of domestic adult cattle were obtained from the local slaughterhouse, sterilized with povidone-iodine solution for 3 to 5 minutes to avoid contamination, and washed with sterile Dulbecco's PBS (DPBS). The harvested corneas were placed epithelial side down in sterilized dishes and incubated in DMEM/F12 with Dispase II at 37°C for 30 to 40 minutes. The loosened epithelial cells were scraped gently using a cell scraper. The cells were washed, seeded onto the cell culture dishes (Corning, NY), and incubated at 37°C in a humidified atmosphere of 95% air/5% CO_2_. The routine culture medium was DMEM/F12 containing 10% FBS, 100 U/mL penicillin, 100 µg/mL streptomycin, 0.25 µg/mL fungizone, 1% insulin-transferrin-selenium supplement and 50 µg/mL gentamicin. The medium was changed every 2 to 3 days.

### Modification of chitosan with 2-iminothiolane

Fifty micrograms of chitosan oligosaccharide lactate (0.28 µEq of NH_2_ group) and 0.341 g 2-iminothiolane were dissolved in 50 mL 0.1 M sodium acetate buffer (pH 6.0) at room temperature. The resulting polymer conjugates were dialyzed in a dialysis membrane bag with a molecular weight cutoff of 3500 Daltons (Spectrum, Philadelphia, PA, USA) against 0.1 M potassium phosphate buffer containing 10 mM DTT at pH 7.4. The degree of modification was determined by quantifying the thiol groups on the modified chitosan with Ellman's reagent [29], [30].

### Modification of Ad with GMBS and chitosan

**Figure 1:**
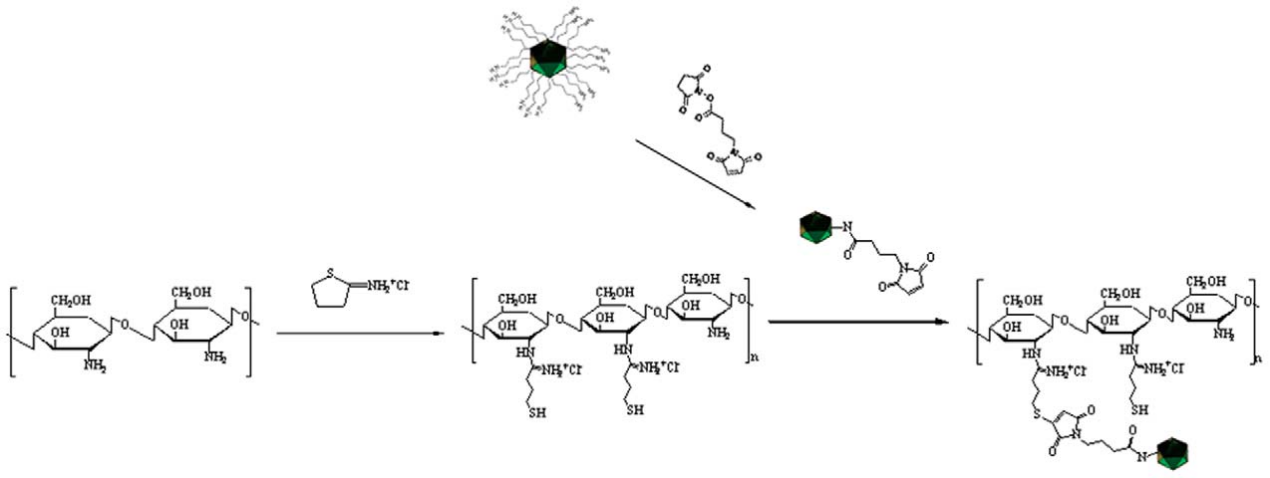
Schematic diagram of covalently modified Ad and chitosan with GMBS and 2-iminothiolane, respectively.

The Ad used in this study was AdCMV-gfp (Qbiogene, Carlsbad, CA), an E1-, E3-deleted Ad5 recombinant expressing green fluorescent protein (GFP) under the control of a cytomegalovirus (CMV) promoter. Because the hexon is the major component of the Ad capsid and is 20-fold more abundant than the knobbed fiber protein in biomedical copy number [31], the lysine residues on the hexon proteins are reasonably considered the major linkage sites of GMBS. Thus, for each µg of purified Ad, 0.38 nEq of viral lysine residues are present, based on the formula: Lysine content = (weight of Ad)×(number of lysine residues in hexon protein sequence)/(molecular weight of hexon protein on viral capsids). Different amounts of the heterobifunctional crosslinking reagent GMBS were conjugated to the Ads with gentle vortexing at room temperature for 30 minute. Subsequently, different amounts of chitosan-SH were mixed with Ad-GMBS, as listed in Table 1, and the mixtures were reacted at room temperature for 2 hours. The sizes and zeta potentials of unmodified Ads and chitosan-modified Ads were determined by a dynamic light scattering (DLS) mthod with a Zetasizer 3000 (Malvern, Worcestershire, UK) [32]. A schematic diagram of covalently modified Ad and chitosan is shown in Fig. 1.

**Table 1:** Comparison of different reaction amounts of GMBS and chitosan-SH to yield different Ad/GMBS/ChiSH ratios.

Reaction mixture	Ad (µg)	Moles of lysine on Ad (nmol)	GMBS in DMSO (µg)	Moles of GMBS (nmol)	ChiSH (µl)	Moles of thiol group (nmol)	Ad/GMBS/ChiSH ratio
I	1	0.38	0.1	0.38	1	2.62	1∶1∶7
II	1	0.38	0.1	0.38	10	26.2	1∶1∶70
III	1	0.38	0.1	0.38	100	262	1∶1∶700
IV	1	0.38	1.0	3.8	1	2.62	1∶10∶7
V	1	0.38	1.0	3.8	10	26.2	1∶10∶70
VI	1	0.38	1.0	3.8	100	262	1∶10∶700
VII	1	0.38	10	38	1	2.62	1∶100∶7
VIII	1	0.38	10	38	10	26.2	1∶100∶70
IX	1	0.38	10	38	100	262	1∶100∶700

### Morphological investigation by transition electron microscopy

Unmodified Ads and chitosan-modified Ads were processed for transmission electron microscopy using a negative stain technique [21]. Ten-microliter drops of the freshly prepared samples were placed on copper grids coated with carbon and formvar (EMS, Washington, PA, USA). The solution was removed with filter paper and replaced with 2% phosphotungstic acid (pH 7.0) for 30 s. After removal of this solution, the grids were allowed to dry and were imaged with a transmission electron microscope (Hitachi, Tokyo, Japan).

### Infection efficiency assay

Bovine corneal epithelial cells were seeded onto a 6-well culture plate (TPP, Trasadingen, Switzerland) at a density of 2×10^5^ cells/well for the infection efficiency assay. After incubation for 24 hours, these cells were transfected with unmodified Ad or a range of chitosan-modified Ads from different reaction mixtures (Table 1) in 2 mL of medium (final volume) with a serial multiplicity of infection (MOI) of 200, 1000 or 5000 Ad particles/cell. To investigate cell infection in the presence of neutralizing antibody, rabbit polyclonal antibody to Ad type 5 was added to the medium at 2 ng/mL. After 24 hours, the cells were harvested, washed with DPBS, treated with TrypLE™ Express, and resuspended in 1 mL of DPBS for flow-cytometric analysis of GFP expression with a FACScan flow cytometer (Becton Dickinson, San Joes, CA). The presence or absence of staining was determined by comparison with the untreated control cells. The data acquisition and analysis were performed using Cellquest software (Becton Dickinson, San Joes, CA). The relative fluorescence intensity of chitosan-modified Ad was defined as the magnitude of the mean fluorescence intensity greater than that of control cells divided by that of the unmodified Ad. In addition, the infected cells were observed and imaged with an inverted microscope (model 1×71, Olympus, Tokyo, Japan) equipped with a digital camera.

### *In vivo* infection

The animal experiments were conducted according to the Guide for the Care and Use of Laboratory Animals and were approved by the National Taiwan University College of Medicine and College of Public Health Institutional Animal Care and Use Committee (IACUC 20060052). All experiments conformed to the ARVO Statement for the Use of Animals in Ophthalmic and Vision Research. To test the ability of chitosan-modified Ads to transfect cells, transgene expression in corneal epithelial cells was evaluated by observing the GFP expression after incubation with Ad-GMBS at a ratio of 1∶100 and Ad-GMBS-ChiSH at a ratio of 1∶100∶700 at 1000 Ad particles/cell. Sprague-Dawley rats, 6 to 8 weeks old and weighing 200 to 250 g, were used in this study, and each group consisted of six animals. Six immune rats had previously been instilled intranasally with 10^9^ infectious units of a replication-defective type 2 adenovirus encoding GFP (AdCMV-gfp) and had titers to Ad in the range of 25,000–50,000. Naive rats had not been exposed to the Ad vector. All animals in the immunized group were subjected to tail vein puncture on the day of instillation, and the blood was analyzed for antibody titers. Titers of neutralization antibodies to AdCMV-gfp in serum preparations were determined by a microneutralization test performed in 96-well microtiter plates with continuous HEK 293 cells, which had been generated by transformation of human embryonic kidney cell cultures. Titers of sera were determined by a neutralization assay on sera serially diluted 2-fold, beginning with a 1∶2 dilution [15], [33]. Diluted AdCMV-gfp with a TCID50 value of 100 was incubated with 2-fold dilutions of sera and inoculated into HEK 293 cells. They were examined after a 72- to 96-hour incubation, and the end point was the highest dilution of sera that inhibited CPE when compared with control tubes containing no antiserum. Data are reported as the reciprocal titer interpolated from duplicate assays. All samples were tested in duplicate.

The contralateral left eye without treatment was used as a control. After topical application for 1 day, eyes were obtained and embedded in gelatin capsules containing Tissue-Tek (OCT Compound 4583; Sakura Fine Tek Europe BV, Zoeterwoude, Netherlands) and frozen in liquid nitrogen. Serial 8-µm-thick cryostat sections were taken across the eyeballs along the optical axis. The transgene expressions were evaluated by fluorescence microscopy (DM L; Leica, Wetzlar, Germany) for Ad-GMBS at a ratio of 1∶100 and Ad-GMBS-ChiSH at a ratio of 1∶100∶700. Both right (treated) and left (control) eyes from each animal were evaluated in duplicate.

## Results

### Characterization of chitosan-modified Ad

In the present study, the covalent attachment of chitosan on Ad was achieved by first modifying chitosan with 2-iminothiolane and cross-linking Ad to GMBS, and subsequently conjugating thiolated chitosan to the GMBS-modified Ad by thioether formation. The amount of 2-iminothiolane immobilized on the chitosan was quantitatively determined using Ellman's method [34]. The degree of modification was 2.62 mEq SH/g chitosan.

**Figure 2:**
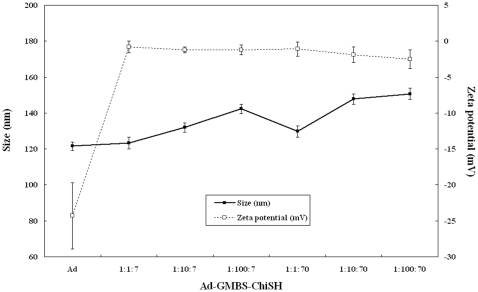
Variations of chitosan-modified Ad at different Ad∶GMBS and Ad∶GMBS∶ChiSH ratios. For size, each point represents the particle number mean ± S.E.M. For zeta potential, each point represents the mean of zeta potential ± S.E.M (n = 100).

**Figure 3:**
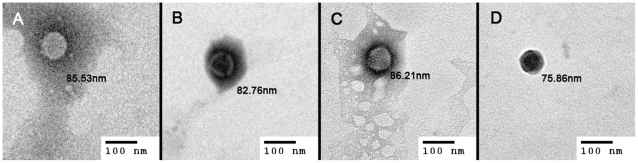
Transmission electron photomicrographs of unmodified Ad (A) and chitosan-modified Ad at Ad∶GMBS∶ChiSH ratios of 1∶1∶7 (B), 1∶10∶7 (C), and 1∶100∶7 (D). Magnification = 80,000×. Scale bar = 100 nm.

Fig. 2 shows the particle sizes and surface charges of chitosan-modified Ads with different Ad/GMBS/ChiSH ratios. When compared to the average size of unmodified Ads (about 120 nm), dynamic light scattering analysis showed that the average size of chitosan-modified Ads gradually increased with increasing amounts of GMBS or/and chitosan. The size distribution of each point was small, excluding the possibility of aggregation. Fig. 2 also shows the surface charge, determined by the measurement of zeta potential, of unmodified and modified Ads. The net surface charge of the Ad capsid changed from −24.3 mV to nearly neutral. In addition, the morphologies of unmodified Ads and chitosan-modified Ads were observed using transmission electron microscopy. Fig. 3 shows the photomicrographs of unmodified Ads and chitosan-modified Ads. The size of chitosan-modified Ad particles increased with the amount of GMBS. Chitosan-modified Ads exhibited a rough and irregular appearance compared to the round shape of unmodified Ads. The morphology of chitosan-modified Ads correlated with the size and size distribution by DLS. The gradual increase in particle size with the amount of GMBS or/and chitosan and neutralization of the viral surface charges may be evidence that the chitosan and Ad were conjugated via GMBS.

### Infection efficiency of chitosan-modified Ad

**Figure 4:**
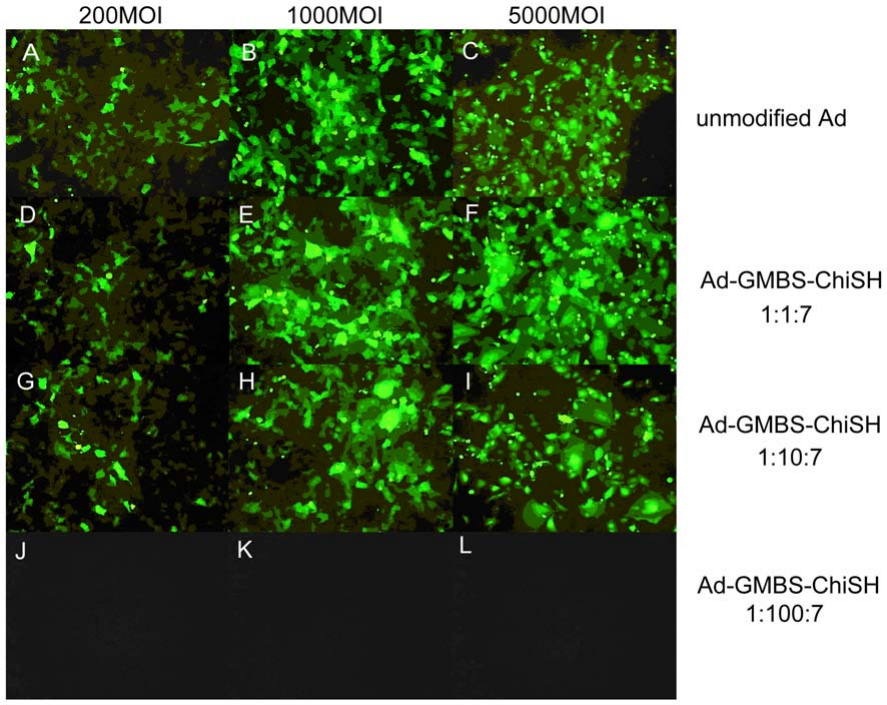
Fluorescence photomicrographs of primary cultured corneal epithelial cells. Primary cultured corneal epithelial cells infected by unmodified Ad (A)–(C) or chitosan-modified Ad at Ad∶GMBS∶ChiSH ratios of 1∶1∶7 (D)–(F), 1∶10∶7 (G)–(I), and 1∶100∶7 (J)–(L). Magnification = 100×.

**Figure 5:**
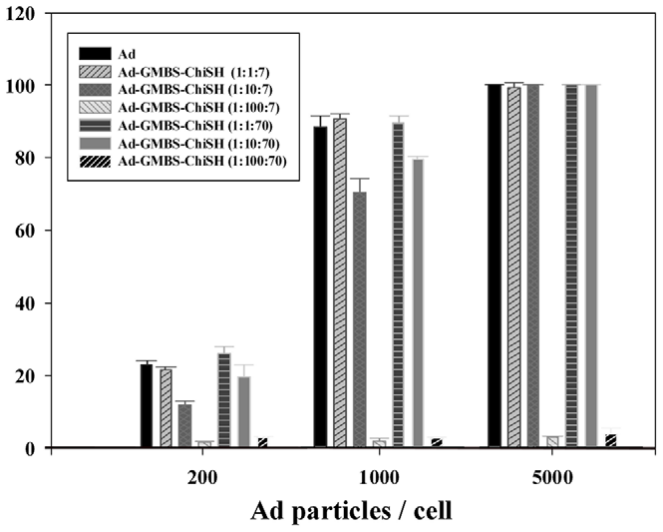
Infection efficiencies from flow-cytometric analysis of corneal epithelial cells infected at a MOI of 200, 1000 or 5000 Ad particles/cell with unmodified Ad or chitosan-modified Ad at varying ratios. Each point represents the mean ± S.D. (n = 3).

To test whether chitosan-modified Ad was able to infect cells and to examine infection efficiency, the expression of GFP was assessed after infecting primary cultured corneal epithelial cells with different GMBS ratios (Fig. 4). Control corneal epithelial cells showed normal phenotypes and no green fluorescence, as expected (data not shown). Flow-cytometric analysis of GFP expression showed that the infection efficiency of chitosan-modified Ads was dose-dependent (Fig. 5). When infected with 1000 Ad particles/cell, corneal epithelial cells infected with chitosan-modified Ads at a 1∶100∶7 Ad/GMBS/ChiSH ratio did not show any detectable fluorescence signal 24 hours after infection, whereas other cells showed bright fluorescence. When infected with 200 Ad particles/cell, the infection efficiency decreased as the amount of GMBS increased. The infection efficiencies of chitosan-modified Ads at 1∶10∶7 and 1∶10∶70 Ad/GMBS/ChiSH ratios were approximately 20–30% lower than that of unmodified Ad. Gene expression of chitosan-modified Ad at 1∶100∶7 and 1∶100∶70 Ad/GMBS/ChiSH ratios fell markedly. Therefore, the ability of Ad to infect corneal epithelial cells was essentially ablated by the modification procedure at higher ratios of GMBS to Ad.

### Chitosan partially restores infection activity

**Figure 6:**
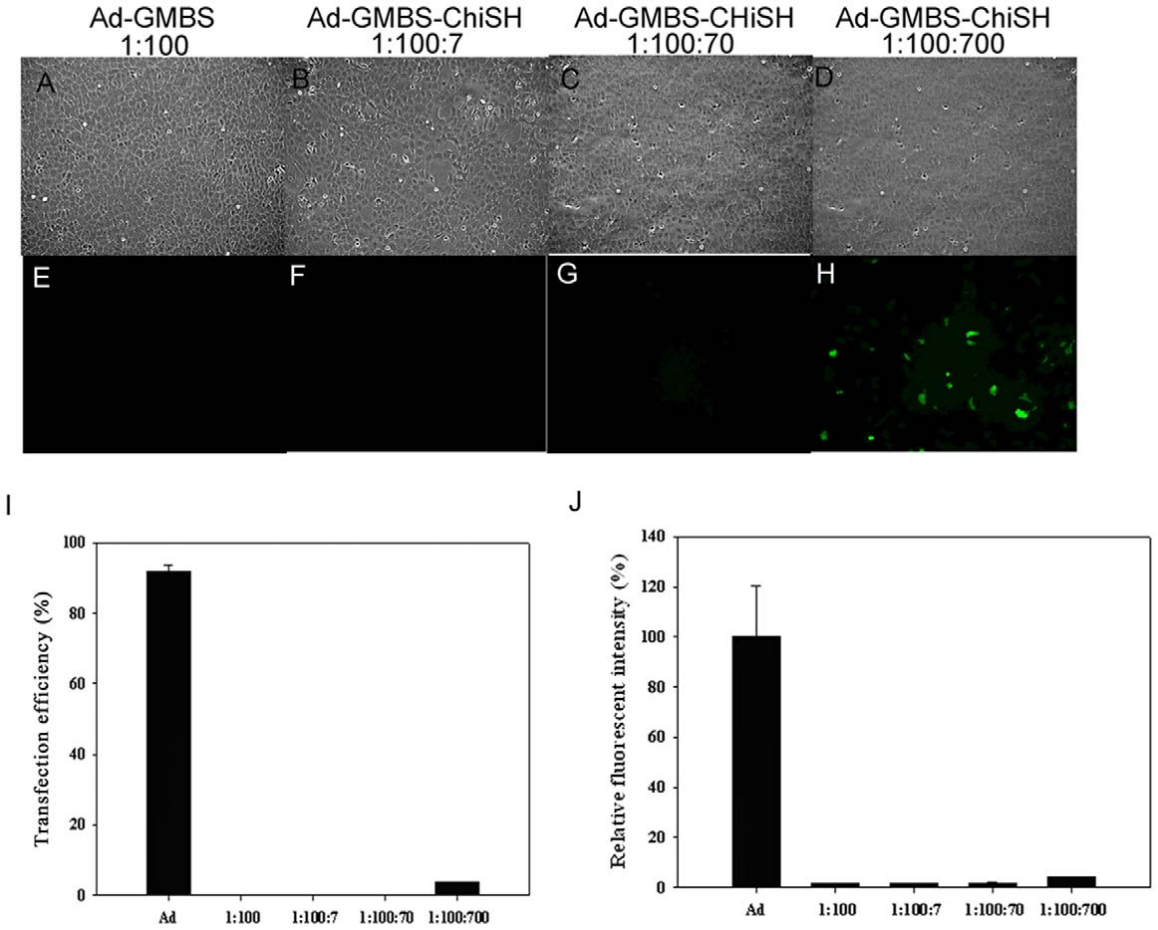
Phase contrast and fluorescence photomicrographs, respectively, of primary cultured corneal epithelial cells infected with Ad-GMBS at a ratio of 1∶100 (A), (E); Ad-GMBS-ChiSH at a ratio of 1∶100∶7 (B), (F); Ad-GMBS-ChiSH at a ratio of 1∶100∶70 (C), (G); and Ad-GMBS-ChiSH at a ratio of 1∶100∶700 (D), (H) at 1000 Ad particles/cell. Magnification = 100×. Infection efficiency (I) and relative fluorescence intensity (J) from flow-cytometric analysis of corneal epithelial cells infected with Ad, Ad-GMBS at a ratio of 1∶100, Ad-GMBS-ChiSH at a ratio of 1∶100∶7, Ad-GMBS-ChiSH at a ratio of 1∶100∶70 and Ad-GMBS-ChiSH at a ratio of 1∶100∶700 at 1000 Ad particles/cell. Each point represents the mean ± S.D. (n = 3). **P<0.001 (Student's t-test).

To determine whether the amount of chitosan could affect the ability of chitosan-modified Ad to transfect cells, transgene expression in corneal epithelial cells was examined after incubation with increasing amounts of chitosan at a 1∶100 Ad-GMBS ratio and 1000 Ad particles/cell (Fig. 6). Cells treated with 1∶100 Ad-GMBS in the absence of chitosan generated 810-fold lower GFP expression than unmodified Ad-treated cells (Fig. 6I). In contrast, cells infected with chitosan-modified Ad expressed GFP in a chitosan-dependent manner. The greatest transgene expression was from Ad-GMBS-ChiSH at a ratio of 1∶100∶700. The infection efficiencies and the mean fluorescence intensities increased with increasing amounts of chitosan (Figs. 6I and 6J). However, the infection efficiencies and the mean fluorescence intensities resulting from transfection with chitosan-modified Ads were still much lower than those of the unmodified Ads. Therefore, higher amounts of chitosan partially restored infection ability.

### Infection efficiency in the presence of neutralizing antibody

**Figure 7:**
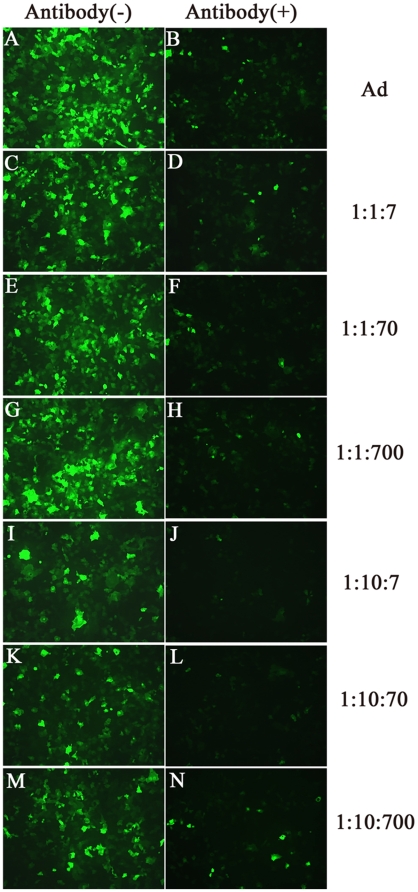
Fluorescence photomicrographs of primary cultured corneal epithelial cells infected with unmodified Ad (A)–(D), Ad-GMBS-ChiSH at a ratio of 1∶1∶7 (E)–(H), Ad-GMBS-ChiSH at a ratio of 1∶1∶70 (I)–(L), Ad-GMBS-ChiSH at a ratio of 1∶1∶700 (M)–(P), Ad-GMBS-ChiSH at a ratio of 1∶10∶7 (Q)–(T), Ad-GMBS-ChiSH at a ratio of 1∶10∶70 (U)–(X) and Ad-GMBS-ChiSH at a ratio of 1∶10∶700 (Y)–(AB) at 1000 Ad particles/cell in the absence or presence of neutralizing antibody. Magnification = 100×.

**Figure 8:**
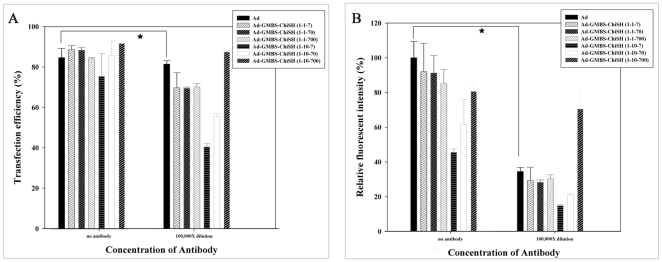
Infection efficiency (A) and relative fluorescent intensity (B) from flow-cytometric analysis of corneal epithelial cells infected with unmodified Ad or chitosan-modified Ad at different ratios at 1000 Ad particles/cell in the absence or presence of neutralizing antibody. Each point represents the mean ± S.D. (n = 3). *P<0.05 (Student's t-test).

Fig. 7 shows the infection efficiencies of unmodified and chitosan-modified Ads in the absence or presence of neutralizing antibody against Ad capsid protein. Neutralizing antibody at 2 ng/mL was sufficient to reduce the infection efficiencies and the relative fluorescence intensities resulting from infection with either unmodified Ad or chitosan-modified Ads, except with Ad-GMBS-ChiSH at a ratio of 1∶100∶700 (Fig. 8). Therefore, the ability of chitosan to evade neutralizing antibodies appeared to work in the present study.

### *In vivo* infection assay

**Figure 9:**
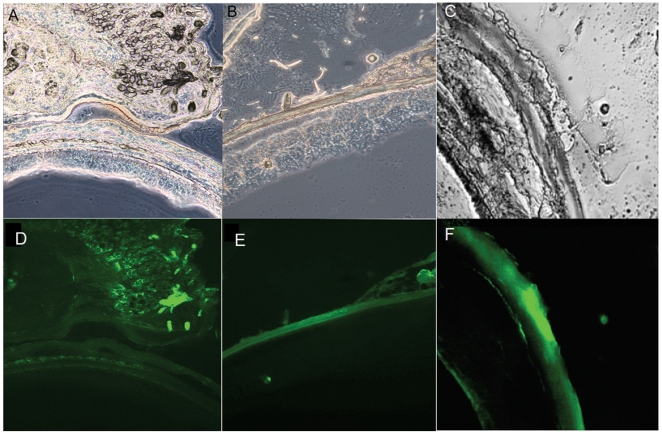
Phase contrast images of corneal samples (A, B and C). Weak fluorescence signals were detected in sections of Ad-GMBS–treated eyes, Magnification = 100× (D). Sections from Ad-GMBS-ChiSH–treated eyes revealed fluorescence localized throughout the corneal and conjunctival epithelial cells. Corneal epithelium from Ad-GMBS-ChiSH–treated eyes was uniformly fluorescent. In the palpebral conjunctiva, fluorescence was evenly distributed along the entire cell and in the bulbal conjunctiva. Magnification = 100× (E). Animals were immunized with Ad vector by intranasal instillation, and serum antibody titers were monitored for 5 weeks. In general, the antibody titers were between 25,000 and 50,000 at the time of challenge with Ad-GMBS-ChiSH (1∶100∶700). Sections from cornea treated with Ad-GMBS-ChiSH (1∶100∶700) revealed fluorescence localized uniformly throughout the corneal epithelial cells. Magnification = 200× (F).

The chitosan-modified Ad and GMBS-modified Ad without further chitosan modification were applied to corneal infection *in vivo*. Fig. 9 shows typical phase-contrast images and fluorescence signals of corneal histology. As expected, sections of Ad-GMBS (1∶100)–treated eyes from two of six rats demonstrated a weak fluorescence signal (Fig. 9D). Sections from Ad-GMBS-ChiSH (1∶100∶700)–treated eyes revealed fluorescence localized throughout the corneal and conjunctival epithelial cells in all six rats. Corneal epithelium from Ad-GMBS-ChiSH–treated eyes was uniformly fluorescent. In the palpebral conjunctiva, it was evenly distributed along the entire cell, as well in the bulbal conjunctiva (Fig. 9E). Rats were immunized with Ad vectors by intranasal instillation, and serum antibody titers were monitored over time. In general, the antibody titers were between 25,000 and 50,000 at the time of challenge with Ad-GMBS-ChiSH (1∶100∶700). The period between immunization and secondary challenge was 5 weeks. Corneal sections treated with Ad-GMBS-ChiSH (1∶100∶700) revealed strong fluorescence localized throughout the corneal epithelial cells uniformly in all six rats (Fig. 9F).

## Discussion

To our knowledge, this is the first report on the modification of the surface of Ad using chitosan to ablate binding between the Ad and cells, to shield the Ad from neutralizing antibodies, and possibly to permit the incorporation of new ligands for retargeting. The possibility to retarget Ad to selected receptors provides the opportunity to redefine its tissue tropism and distribution *in vivo*. The avoidance of Ad infection in nontarget tissues would decrease the viral load. Evasion of neutralization by pre-existing anti-Ad antibodies can also decrease the viral load. Decreasing the viral load may lower toxicity associated with Ad gene therapy and improve its efficacy and safety.

The cationic property of chitosan is responsible for ionic interactions with anionic substructures such as sialic acid and sulfonic acid within the mucus layer, thereby conferring its mucoadhesiveness [23]. In addition, chitosan exhibits favorable biological behavior, such as permeability-enhancing properties, and interesting physicochemical characteristics that make it a unique material for the design of ocular delivery [23], [35], [36], [37], [38]. Previous studies have demonstrated enhanced Ad infectivity using a noncovalent complex consisting of chitosan [25], [26]. One of the mechanisms proposed for this enhanced effect is that cationic chitosan forms a complex with negatively charged Ad, and excess positive charges in the Ad and chitosan-containing complex enhance interactions with negatively charged cell surfaces through electrostatic interactions. This phenomenon is consistent with our data showing that greater amounts of chitosan restored infection ability (Fig. 5). Therefore, we expect that the infection route of chitosan-modified Ad into cells may occur via a receptor-independent pathway.

In addition, previous studies using monovalent PEG or the multivalent polymer pHPMA for conjugation to the surface of Ads have been reported [11]–[16], [21], [22], [39]–[41]. These modifications have demonstrated that the loss of Ad bioactivity depends on the degree of modification of viral lysine residues [11], [14], [21]. We obtained similar results, as the infection efficiency of chitosan-modified Ad decreased with increasing GMBS (Fig. 4). It is reasonable to attribute the decreased infection efficiency after modification of viral lysine residues to interference of the interaction between Ad and the cells through steric hindrance. On the other hand, the new physical characteristics of the chitosan-modified Ad may also contribute to the observed viral infection results. Zeta potential measurements have shown that Ad capsids bear a significantly negative charge. The surface charges of viral vectors can significantly affect the level of infection [42], [43]. Adenoviral infection is inhibited due to static repulsion between the negatively charged sialic acid residues on the cell surface and the Ad [44]. Positively charged chitosan-SH effectively masks and neutralizes the groups responsible for the repulsive charge and produces an environment that favors nonspecific interaction of the virus with the cell membrane.

The infection efficiencies of the unmodified Ads and chitosan-modified Ads at different ratios (1∶1∶7, 1∶1∶70, 1∶10∶7 and 1∶10∶70) depended on the number of virus particles in the medium (Fig. 3). Accordingly, the transgene expression depends on the number of Ad particles entering cells. However, the infection efficiencies of chitosan-modified Ad at ratios of 1∶100∶7 and 1∶100∶70 were not significantly greater than those of control cells. This indicates that chitosan-modified Ad at ratios of 1∶100∶7 and 1∶100∶70 could not successfully enter cells due to insufficient amounts of chitosan to mask the negatively charged Ad, which have already lost their innate infection ability.

Ultimately, the goal of gene therapy is to transfer genes to tissues and to affect pathophysiological processes. The topical application of viral vectors takes advantage of the superficial location of the corneal epithelium and requires minimally invasive techniques. The integrity of the corneal epithelial layer has consistently been found to profoundly reduce the efficiency of gene transfer. Specifically, an intact epithelium is a poor substrate for gene therapy. Attempts to transfer markers or biologically active genes by the direct application of adenoviral vectors have demonstrated poor epithelial gene expression. The precise mechanism for this apparent resistance of intact epithelium is not yet understood; however, the presence of superficial ocular surface barriers (i.e., tear film constituents) or the relatively low rate of cell division of intact epithelium may play a role [45]. Furthermore, chitosan-coated nanocarriers have a great affinity for corneal and conjunctival epithelial cells [35]. Similarly, nanoparticles made solely of chitosan exhibit a facilitated interaction with the cornea and conjunctiva, where they remain for more than 24 hours [38]. Based on these observations, we presume that chitosan-modified Ad may benefit from some of these advantages, and we applied chitosan-modified Ad in the right eye every 30 minutes for 6 hours to increase the infectious efficiency in the ocular surface. Although the efficiency of the developed reagent is poor and requires long exposure, our method is able to function in an *in vivo* system to increase the infection possibilities as compared to previously employed methods.

We also carried out experiments on rats immunized with Ad vectors by intranasal instillation, and serum antibody titers were monitored over time until the antibody titers were between 25,000 and 50,000 at the time of challenge with topical Ad-GMBS-ChiSH (1∶100∶700). The period of time between immunization and secondary challenge was 5 weeks. The corneal tissues were then harvested and cryosectioned. We found that corneal sections treated with Ad-GMBS-ChiSH (1∶100∶700) revealed strong fluorescence localized throughout the corneal epithelial cells uniformly in all six rats (Fig. 9F). Our data support the idea that the covalently conjugated Ad is resistant to neutralizing anti-Ad antibodies and could be used to express a transgene in the ocular surface. Compared to PEG-modified Ad [15], chitosan-modified Ad at a ratio of 1∶10∶700 (Ad∶GMBS∶ChiSH) protected viruses from antibody neutralization at a low concentration. We reason that, unlike PEG, chitosan has a rigid structure of hexose to allow chitosan modification to effectively protect the virus at a lower concentration of neutralizing antibody.

In conclusion, chitosan can provide a great platform for chemical modification of Ad. It permits the incorporation of a range of targeting molecules and other biological effectors. Therefore, chitosan-modified Ad provides promising potential for further *in vivo* studies.
